# Supplementary material for: Effect of core training on athletic and skill performance of basketball players: A systematic review
Source: PLoS One. 2023 Jun 22;18(6):e0287379. doi: 10.1371/journal.pone.0287379 (PMC10286970; doi:10.1371/journal.pone.0287379)
Supplement: Attachment — Submitted filename: Response to Reviewers.docx [file pone.0287379.s003.docx]

June 1, 2023

Donald L. Hoover

Academic Editor

*PLOS ONE*

=============

**Dear Prof. Donald L. Hoover**

We would like to resubmit our revised manuscript for consideration of publication in *PLOS ONE*. The manuscript is entitled “Effect of Core Training on Athletic and Skill Performance of Basketball Players: A Systematic Review.” PONE-D-22-29930

The authors deeply appreciate the very detailed review of the manuscript and all the thoughtful comments that will undoubtedly help to improve it substantially. We have studied comments carefully and have made corrections which we hope meet with approval. Revised portions are made as track-change in the manuscript. The following is a point-by-point response to the reviewers’ comments. We hope that our manuscript can now be considered for publication in “*PLOS ONE*”.

Comments from the editors and reviewers:

Dear editors /referees many thanks for your constructive and valuable criticisms. Our responses are presented below and we are looking forward and ready to respond to any future comment (s)

Journal Requirements:

Journal Requirements:

Please review your reference list to ensure that it is complete and correct. If you have cited papers that have been retracted, please include the rationale for doing so in the manuscript text, or remove these references and replace them with relevant current references. Any changes to the reference list should be mentioned in the rebuttal letter that accompanies your revised manuscript. If you need to cite a retracted article, indicate the article’s retracted status in the References list and also include a citation and full reference for the retraction notice.

**Response: Thank you for your insightful comment. We checked the reference list to ensure that it is complete and correct.**

Additional Editor Comments (if provided):

The authors have done a nice job of addressing reviewer suggestions and revising this manuscript. The re-review created a few more minor suggested edits. The authors are encouraged to make these revisions in a timely manner and then resubmit.

**Response: Thank you very much for your encouraging comments and for recommending our manuscript. We are pleased to address all the reviewer’s comments as presented below. We hope our response meets your acceptance criteria.**

Reviewers' comments:

Reviewer's Responses to Questions

**Comments to the Author**

1. If the authors have adequately addressed your comments raised in a previous round of review and you feel that this manuscript is now acceptable for publication, you may indicate that here to bypass the “Comments to the Author” section, enter your conflict of interest statement in the “Confidential to Editor” section, and submit your "Accept" recommendation.

Reviewer #1: (No Response)

2. Is the manuscript technically sound, and do the data support the conclusions?

The manuscript must describe a technically sound piece of scientific research with data that supports the conclusions. Experiments must have been conducted rigorously, with appropriate controls, replication, and sample sizes. The conclusions must be drawn appropriately based on the data presented.

Reviewer #1: Yes

3. Has the statistical analysis been performed appropriately and rigorously?

Reviewer #1: Yes

4. Have the authors made all data underlying the findings in their manuscript fully available?

The PLOS Data policy requires authors to make all data underlying the findings described in their manuscript fully available without restriction, with rare exception (please refer to the Data Availability Statement in the manuscript PDF file). The data should be provided as part of the manuscript or its supporting information, or deposited to a public repository. For example, in addition to summary statistics, the data points behind means, medians and variance measures should be available. If there are restrictions on publicly sharing data—e.g. participant privacy or use of data from a third party—those must be specified.

Reviewer #1: (No Response)

5. Is the manuscript presented in an intelligible fashion and written in standard English?

PLOS ONE does not copyedit accepted manuscripts, so the language in submitted articles must be clear, correct, and unambiguous. Any typographical or grammatical errors should be corrected at revision, so please note any specific errors here.

Reviewer #1: Yes

6. Review Comments to the Author

Please use the space provided to explain your answers to the questions above. You may also include additional comments for the author, including concerns about dual publication, research ethics, or publication ethics. (Please upload your review as an attachment if it exceeds 20,000 characters)

Reviewer #1: Thank you for the opportunity to re-review this article, which bridges addresses the topic of core stability training and its potential impact upon sport-specific performance measures within the sport of basketball. As I noted in my original review, this topic has received little attention in the scientific literature. Additional information on this topic may be helpful for sport-specific coaches, conditioning coaches, and sport scientists. In sum, I believe the authors have adequately addressed my suggestion for better explaining the “why” behind the mechanisms that may contribute to this functional carryover seen with core training.

As noted in my original review, I see this manuscript as generally well-written and clearly laid out. In the revision, the authors have done a fine job of integrating my original suggestion regarding the inclusion of motor behavior theory and how it may reasonably illustrate the conceptual rationale for how this type of core training may carry over to increased functional performance on sport-specific skills within the sport of basketball. The value of this cannot be overstated, given the goal of all coaches to make links between the training they engage athletes in and the carryover to on-court performance.

Otherwise, my suggestions for improving this manuscript are largely related to “cleaning up” a few additional English-usage issues. In re-reading this manuscript, I have found a few junctures where I believe the word usage can be improved, so I’ve made some specific friendly edits toward the goal of contributing to the overall “readability” of the manuscript.

In summary, the aim of this project has merit. I recommend the authors address the issues noted below prior to this manuscript appearing in this peer-reviewed journal. Thanks once again for the opportunity to review this paper.

**Response: Thank you very much for your encouraging comments and we are pleased to respond. Thank you for recommending our manuscript.**

Specific comments:

Lines 59-60: Suggest revising to the following: “European and American experts began to widely implement strength training principles in a variety of sports in the late 1990s.”

**Response: Thank you for your careful review. We have carefully considered your valuable suggestions and revised the sentence (lines: 59-61).**

Line 116: Need a citation here, at the end of this sentence. The Magill textbook will work fine here (29).

**Response: Thank you for your insightful comment. We included the suggested reference (line 116).**

Line 127: Suggest adding the following sentence to the end of this paragraph: “In sum, the specificity hypothesis is likely helpful in understanding in what ways core strength training may contribute to improved sport-specific performance.”

**Response: Thank you for your careful review. We have carefully considered your valuable suggestions and revised the sentence (lines: 127-129).**

Line 138: Suggest a minor revision as follows: “This systematic review aims to elucidate the impact of core training as a function of the above-noted specificity hypothesis on basketball players’ physical and skill performance.” Restating in this manner will help to better tie in the new content the authors have included regarding the general vs specificity concept, thus helping to better elucidate why they have elected to do this study.

**Response: Thank you for your careful review. We have carefully considered your valuable suggestions and revised the sentence (lines: 139-141).**

Line 134: Need to correct this typo. I assume it’s referring to VO2 max but not clear as written.

**Response: Thank you for your careful review. We have corrected this typo (line 136).**

Line 166: Revise to the following: “…addressing the impact of at least one core strength intervention on…”

**Response: Thank you for your careful review. We have carefully considered your valuable suggestions and revised the sentence (lines: 163-164).**

Line 257: Replace “are” with “were”

**Response: Thank you for your careful review. We have carefully considered your valuable suggestions and revised this word (line 251).**

Line 341: Suggest revising to: “There is a lack of consensus on whether an athlete should use strength training within a sport-specific training program, what type of strength training should the athlete use, when to use, and how to apply it.” Citations are needed for this sentence; number 7 likely works here but others are needed too.

**Response: Thank you for your careful review. We have carefully considered your valuable suggestions and revised the sentence (lines: 334-336). We also included the recommended citation and two other ones.**

Gabbett TJ. The training-injury prevention paradox: should athletes be training smarter and harder?. Br J Sports Med. 2016;50(5):273-280. doi:10.1136/bjsports-2015-095788

Granacher U, Lesinski M, Büsch D, et al. Effects of Resistance Training in Youth Athletes on Muscular Fitness and Athletic Performance: A Conceptual Model for Long-Term Athlete Development. Front Physiol. 2016;7:164. Published 2016 May 9. doi:10.3389/fphys.2016.00164

Line 345: Need a citation here, at the end of the sentence. Also, suggest revising to “General strength training can provide overall protective strength ….”

**Response: Thank you for your careful review. We have carefully considered your valuable suggestions and revised the sentence (lines: 338-339). We also included a citation as recommended.**

Hughes DC, Ellefsen S, Baar K. Adaptations to Endurance and Strength Training. Cold Spring Harb Perspect Med. 2018 Jun 1;8(6):a029769. doi: 10.1101/cshperspect.a029769. PMID: 28490537; PMCID: PMC5983157.

Line 346: Suggest revising to: “Adding additional muscle can also help with energy transfer, as a mix of fast and slow twitch fibers can provide more energy uptake, which can help athletes with endurance [45]”.

**Response: Thank you for your careful review. We have carefully considered your valuable suggestions and revised the sentence (lines: 342-344).**

Line 348: Suggest revising to: “To build any type of muscle, however, the muscle must be overloaded, which causes fatigue and necessitates recovery.”

**Response: Thank you for your careful review. We have carefully considered your valuable suggestions and revised the sentence (lines: 346-347).**

Line 350: Suggest revising to: “Furthermore, most coaches, trainers, and sport scientists understand that most athletes do not possess unlimited hours each day to train nor do they have limitless capacity to recover from training and competition, so they must be highly strategic in what they include in training plans [46,47].”

**Response: Thank you for your careful review. We have carefully considered your valuable suggestions and revised the sentence (lines: 349-352).**

Line 352: Suggest revising to: “As a result, highly specific strength training appears to be most beneficial to athletes, as it can improve the execution of sports activities, particularly as external resistance may be added to make the training task more difficult. Specific strength training can stimulate focused muscle growth, increasing further power, endurance, and speed of movement without requiring the excessive time commitments or undue fatigue that generalized strength training programs may be likely to generate [48]”
**Response: Thank you for your careful review. We have carefully considered your valuable suggestions and revised the sentence (lines: 354-359).**

**====================================================================**

**Finally, we would like to express our gratitude to the editors and the reviewers for their time, efforts and their valuable comments which helped us improve our manuscript.**

**Best regards;**

**Yanmei Zhao**

[**cherryxf99@yxnu.edu.cn**](mailto:cherryxf99@yxnu.edu.cn)

**Corresponding author**
